# Appropriate Use Criteria for Coronary Angiography at Two Hospitals in Southern Brazil: "Doing the Right Things And Doing Things Right"

**DOI:** 10.5935/abc.20190084

**Published:** 2019-05

**Authors:** Marco A. Magalhaes, Jamil R. Cade

**Affiliations:** 1 Hospital Santa Marcelina - Cardiologia Intervencionista, São Paulo, SP - Brazil; 2 Faculdade Santa Marcelina - Escola de Medicina, São Paulo, SP - Brazil

**Keywords:** Coronary Artery Disease/diagnostic imaging, Coronary Angiography, Síndrome Coronariana Aguda, Percutaneous Coronary Intervention/methods, Epidemiology

Cardiovascular disease and particularly coronary artery disease (CAD) remain a global health issue,^1^ notwithstanding major advances in cardiovascular care that have resulted in a reduction in CAD mortality over the last decades.^2^ Indeed, the most recent trends point towards a bottoming out of CAD mortality rates and, for certain subgroups, rates might even be increasing.^3^ The reasons for these alarming trends relate to the prevalence of risk factors, health care system failures in dealing with chronic diseases, unequal access to technology, decreasing levels of investment in cardiovascular research, and persistent heterogeneity in the quality of care.^4^

In contrast, cardiovascular health care costs continue to follow a linear upward trend.^5^ As a consequence, the delivery of high-value cardiovascular care has been reduced.^6^ In the long run, cardiovascular research and innovation should stimulate the development of novel drugs and therapies. In the short term, for struggling health care systems facing escalating costs, avoiding inappropriate tests and ineffective therapies is part of a value-based healthcare agenda.^7,8^ The fundamental concept of moving from volume to value may mitigate conflicting expectations among payers, providers, patients and physicians, who should share a common objective of reducing unwarranted health costs while improving outcomes.^9^

However, it is time for this agenda to be transformed from discussion into action. Taking the lead in this transformation over the last decade, physicians representing the North American medical societies have come together to provide evidence-based recommendations and expert opinions for a range of diagnostic and therapeutic procedures. These evolving recommendations, namely Appropriate Use Criteria (AUC), aim to assist physicians in providing high-value cardiovascular care.

In this issue of Arquivos Brasileiros de Cardiologia, Luciano et al.^10^ present results on the use of AUC for diagnostic catheterization (DC) in Brazil.^10^ From May to October 2016, data were obtained for DC performed at two tertiary hospitals (a general hospital vs. a cardiology hospital). The authors collected data that allowed appropriateness scores to be assigned for each DC, namely, "appropriate" (7 to 9), "occasionally appropriate" (4 to 6) or "rarely appropriate" (1 to 3). Of note, according to the original AUC, the same scoring system was used, but the descriptive terms used were "appropriate", "uncertain" and "inappropriate", respectively.^11^ Additionally, the authors compared each of the three AUC categories between hospitals and with the presence of CAD. The presence of obstructive CAD was defined as angiographic obstruction of more than 50% in the left main coronary artery or 70% elsewhere.

The sample included 737 DC in patients with a mean age of 62 years. Taken together, 80.6% of the exams were deemed appropriate according to AUC criteria, and 15.1% occasionally appropriate (uncertain), while 4.3% were rarely appropriate (inappropriate). Among similar studies, the rates of appropriate, uncertain and inappropriate use of diagnostic catheterization were 52.8%, 31% and 10.8% in Ontario, and 35%, 40% and 25% in New York, respectively.^12,13^ Notably, in the Brazilian study, the rate of inappropriate use in stable patients only (~10.1%) was similar to that in the Ontario cohort (10.8%) and roughly two-fold lower than in the US study. Interestingly, both Canada and Brazil have public universal health systems, which differ from that in the United States, where the health system is predominately financed by private funds.^9^

The second finding that deserves careful attention is the lack of severe obstructive CAD in 41.2% of DC. Although this frequency is lower than that in the Canadian cohort (54.5%), it stills represents an important source of cardiovascular expenditure that can be improved through a comprehensive and specialized assessment (pre-test probability). Indeed, the frequency of normal DC findings was significantly lower at the specialized cardiology hospital than at the general hospital (37.8% vs. 52.6%; p = 0.008), despite a three-times higher volume at the former. Moreover, among patients under CAD investigation, the rates of appropriate DC were significantly higher at the cardiology hospital compared with the general hospital (87.3% vs. 58.5%; p < 0.01). Therefore, these results constitute indirect evidence of higher quality performance in high-volume and specialized centers of cardiovascular care.

There are a number of caveats relating to the study. First, the sample size was relatively small. Second, the AUC categorization was made by the same non-blinded physician who performed the DC and participated in the final decision on whether to proceed with intervention. Third, neither baseline risk factors nor stress test findings were reported, particularly for the general hospital that had a higher proportion of CAD investigation (stable patients) compared with cardiology hospital (73% vs. 34%) Fourth, there was a lack of functional and intravascular invasive imaging assessments. Finally, the sample included only patients from the public health system and clinical outcomes were not presented.

A further interesting finding of Luciano et al.^10^ relates to the reasons behind the rarely appropriate (inappropriate) category of DC and the decision-making upstream. The higher the frequency of inappropriate DC, the more likely the frequency of further inappropriate interventions, a phenomenon called the "diagnostic-therapeutic cascade".^14^ The danger of this cascade was averted in the two Brazilian hospitals, however, where ALL patients rated as receiving an inappropriate DC, 21.9% of whom had severe obstructive CAD, remained under clinical treatment, which was carried out according to the best evidence available. We commend the authors and physicians for "doing the right things AND doing things right", thus benefiting patients and the health care system.
